# Editorial: Advances in brain tumors diagnosis and treatment

**DOI:** 10.3389/fmed.2023.1152547

**Published:** 2023-05-04

**Authors:** Dario de Biase, Enrico Franceschi, Gianluca Marucci

**Affiliations:** ^1^Department of Pharmacy and Biotechnology (FaBiT), University of Bologna, Bologna, Italy; ^2^Solid Tumor Molecular Pathology Laboratory, IRCCS Azienda Ospedaliero-Universitaria di Bologna, Bologna, Italy; ^3^Nervous System Medical Oncology Department, IRCCS Istituto Delle Scienze Neurologiche di Bologna, Bologna, Italy; ^4^Neuropathology Unit, Fondazione IRCCS Istituto Neurologico Carlo Besta, Milan, Italy

**Keywords:** brain tumors, gliomas, brain tumors diagnosis, molecular pathology of brain tumors, microRNA, target therapy, therapeutic approach in brain tumors

The 2021 WHO (World Health Organization) classification of Central Nervous System (CNS) tumors has integrated the histological findings with molecular characterization (1). The characterization of brain tumors to predict survival outcomes and treatment response has been significantly improved (2, 3). This Research Topic focused on the application of novel discovery in characterizing brain tumors for diagnostic, prognostic, or predictive purposes and draws together a series of reports focusing on different aspects of this important item.

## New tumor types and rare entities

The 2021 WHO classification of CNS tumors recognized various new tumor types. PLNTY (Polymorphous low-grade neuroepithelial tumor of the young) is a novel epileptogenic neoplasm, included in the 2021 WHO classification (1). Fei et al. reported 8 new cases of PLNTY describing its clinical, histopathological, imaging, and molecular profile. The authors observed that all cases exhibited intense labeling of CD34 and the absence of *IDH1*/*IDH2* mutations and 1p/19q codeletion. The *BRAF* p.V600E mutation was detected in 66.7% of cases. In this paper, the authors demonstrated that the post-operative seizure-free rate is improved by early surgical intervention and enlarged resection of epilepsy-associated PLNTY.

The skull-base meningiomas with extracranial extensions are a rare type of meningiomas. He et al. summarized the prognostic and clinical features of skull-base meningiomas with extracranial extensions. About 50 cases of skull-based meningiomas were investigated to review clinical symptoms, treatment strategies, radiological characteristics, and prognosis. Patients with communicative meningiomas were younger when compared with those with intracranial meningiomas. Moreover, these patients showed a higher tendency to develop low-grade tumors. Imaging allowed the detection of high bone invasion rate, heterogeneous enhancement, high dural tail sign rate, and high incidence of peritumoral edema.

Internò et al. overviewed the role of molecular aberrations in astrocytomas together with the most effective post-surgical strategies. According to their data, the subgroup of *IDH1*/*IDH2* mutant astrocytomas showed a demonstrable survival benefit with adjuvant chemoradiotherapy, if compared to the *IDH*-WT group.

## Prediction of post-operative mortality

The relationship between post-operative mortality in patients undergoing craniotomy and pre-operative blood urea nitrogen (BUN) was investigated by Liu Y. et al.. The study showed that post-operative mortality was associated with pre-operative BUN. Data demonstrated that the risk of post-operative mortality may be reduced by proper pre-operative management of BUN.

## Radiotherapy in patients with glioblastoma

Chemoradiation followed by maintenance temozolomide (TMZ) with tumor-treating fields (TTFields) is a treatment for patients with newly diagnosed glioblastoma (GBM) (4). Miller et al. evaluated the safety of chemoradiation used concurrently with TTFields. The Authors evidenced that concurrent scalp-sparing chemoradiation with TTFields is a well-tolerated and feasible treatment option, with only limited toxicity.

Huang Y. et al. proposed an approach to delineate the clinical target volume in GBM, based on the relationship between the neural pathways and the growth patterns, enrolling a total of 69 patients. The clinical target volume (CTV) was delineated using a new approach. The used regimen showed a trend of lower rates of marginal recurrence, and the brain volume of high-dose radiation fields was similar to that of the European Organization for Research and Treatment of Cancer (EORTC).

Kaina et al. discussed the synergistic effect of temozolomide and radiation, proposing an optimal timing of TMZ treatment concurrent with radiotherapy. The authors assert that it could be concluded that the TMZ treatment should initially be carried out for 3 days without radiotherapy.

## Immunotherapies in high-grade gliomas

The development of immunotherapies in the treatment of High-Grade Gliomas (HGG), has been limited by several elements, such as the anti-inflammatory tumor microenvironment (TME) (5). Franson et al. reviewed in depth the TME in HGG (adult and pediatric type), the various drivers of the TME in HGG, and different immunotherapeutic approaches. Namely, they focused on myeloid-derived suppressor cells (MDSCs), glioma-associated macrophages and microglia (GAMs), T cell infiltration and dysfunction, cytokines as a putative driver of tumor immune escape, *IDH* mutations, and effects on the TME, the actual immunotherapies and the challenges in immunotherapy development for HGG.

Immunotherapy may become a promising approach also for GBM treatment (6), even if the role of PD-1/PD-L1 expression in GBM is still controversial. Guo et al. performed a meta-analysis to verify the possible link between high/positive PD-L1 expression and overall survival (OS) in GBM. According to this study, in GBM there is a positive correlation between PD-L1 and low OS.

Inflammation is a crucial marker to promote tumorigenesis and tumor progression. However, the role of immune-related genes in GBM remains still unclear. Yu et al. looked for a relationship between the immune microenvironment and GBM. The Authors examined GBM-related RNA sequencing (RNA-seq), clinical data, and survival, acquiring data from four databases. According to their study, three immune-related genes (*PTX3, TNFSF9*, and *BMP2*) may be prognostic factors for patients with GBM.

## Brain metastases

CNS metastases are the most common brain tumor type in adults. The extent of resection and the impact of post-operative residual tumor burden (RTB) in brain metastases are still not defined enough. In the study by Aftahy et al. the authors demonstrated that RTB is a predictor for survival.

Barakeh et al. performed NGS (Next-Generation Sequencing) on brain metastases from the following primary tumors: breast carcinomas, colorectal cancers, renal, and thyroid tumors. The Authors identified clinically relevant mutations in brain metastases that were not detected in the corresponding primary tumors, as alterations in the MAPK, PI3K, and CDK pathways. These data highlighted the possibility of having differences between brain metastases and primary cancers regards molecular profile, and thus the importance of performing the molecular analysis on brain metastatic samples for further clinical management.

Leptomeningeal metastases (LM) of lung cancer have been usually associated with poor prognosis. Osimertinib has shown promising efficacy in NSCLC–LM patients, however resistance to osimertinib develops over time. Yang et al. evaluated the clinical effects of *EGFR* amplification by targeted next-generation sequencing in Cerebrospinal fluid (CSF) samples in patients diagnosed with NSCLC–LM and who had received previous EGFR-TKI treatment. *EGFR* amplification had been evaluated by targeted next-generation sequencing in Cerebrospinal fluid (CSF) samples. Data suggested that the amplification of the *EGFR* gene may induce resistance of NSCLC–LM patients to EGFR-TKIs.

Tumor cell infiltration at the macro-metastasis/brain parenchyma interface (MMPI) is usually correlated with poor outcomes. Blazquez et al. identified specific magnetic resonance imaging (MRI) patterns in patients with brain metastasis, and correlated patient outcomes with these MRI patterns. The authors analyzed the preoperative magnetic resonance images of about 260 patients with brain metastasis. Their results indicated that the MRI breakout pattern is an imaging biomarker for particularly poor outcomes in patients with brain metastasis.

## New diagnostic technologies

New approaches are increasingly leveraged to better understand complex biological systems. The tricarboxylic acid (TCA) cycle is related to the occurrence and development of glioma, and cuproptosis is closely related to the inhibition of the TCA cycle (7). Ye et al. developed and validated cuproptosis-associated prognostic signatures in WHO 2/3 gliomas. In their study, the authors found that eight cuproptosis-related genes (CRGs) were differentially expressed between glioma and normal tissues. The Authors constructed a cuproptosis-associated risk signature, able to predict the prognosis of glioma patients.

Ferroptosis, caused by excessive lipid peroxidation, is another form of form cell death (8). Huang Q. R. et al. have explored the prognostic value of ferroptosis-related lncRNAs (long non-coding RNAs) in low-grade glioma (LGG). Three databases (The Cancer Genome Atlas—TCGA, Chinese Glioma Genome Atlas—CGGA, and Gravendeel) were used to obtain the expression profiles. The analysis led to build a risk signature consisting of 8 lncRNAs, with a good performance in predicting the prognosis of LGG.

Necroptosis is a programmed inflammatory cell death or lysis cell death, playing a fundamental role in killing damaged cells and/or pathogen-infected (8). Xia et al. analyzed the differentially expressed necroptosis-related lncRNAs and their possible impact on the overall survival of glioma patients. The risk score model developed by the authors according to nine necroptosis-related lncRNAs allowed the prediction of the prognosis of glioma patients.

The detection of circulating tumor cells (CTCs) is a promising technology in tumor management (9). A karyoplasmic ratio (KR)-based identification method was developed by Zhu X. et al.. An automatic recognition algorithm was constructed, to determine the correlation between patients' clinical characteristics and high-KR circulating tumor cells. This study revealed a correlation between CTCs and patients' clinical characteristics and increased the efficiency of detecting glioma CTCs.

Liquid biopsy is a minimally invasive method of analysis of molecular biomarkers (e.g., circulating tumor cells, cell-free DNA) starting from any type of patient's body fluid (e.g., plasma, urine, bile, pleural effusion, and cerebrospinal fluid). The lack of permeability of the blood-brain barrier (BBB) may limit the release of ctDNA into the blood. However, it has been shown that exosomes microvesicles and apoptotic vesicles can all cross the intact BBB, and then peripheral blood may be sometimes used for analysis of circulating markers in glioma (10). Balana et al. reviewed several studies using radiogenomics and liquid biopsy in the characterization of gliomas. The authors demonstrated that liquid biopsies and radiogenomics will likely be used initially for additional diagnostic information that could be incorporated into routine clinical practice in the future.

Micro-RNAs are one of the circulating markers that can be analyzed in peripheral blood in patients with gliomas (11). Xu et al. have studied the potential clinical role of miR-4297 in glioma. The authors observed that miR-4297 levels were higher in females with high-grade glioma, but not in male patients, and a higher level of miR-4297 had been associated with a higher risk of glioma recurrence.

In the study by Liu X. et al. specific urine metabolites of Medulloblastoma (MB) were identified using liquid-chromatography–mass spectrometry (LC–MS)-based metabolomics. MB was distinguished with high diagnostic accuracy from non-MB by the combination of cortolone and tetrahydrocortisone, demonstrating that urine metabolomics might be also used for MB monitoring.

Based on the recent tumor classification, a comprehensive approach to molecular testing in the adolescents and young adults (AYA; aged 15–39) was proposed by Lim-Fat et al.. This review will help in improving the classification and identification of brain tumors in the AYA population.

Liu D. et al. explored tumor habitat characteristics in the peritumoral and intratumoral regions, to help in distinguishing primary central nervous system lymphoma (LMPA), common malignant brain tumors, and brain metastases. Quantitative radiomics features provide a useful tool for the non-invasive assessment of a CNS tumor. The model classifier can preoperatively differentiate GBM from brain metastases and LMPA by incorporating peritumoral information into the model.

High-grade glioma (HGG) and primary central nervous system lymphoma look similar under imaging. Zhang et al. performed a systematic metanalysis to determine the efficacy of 18F-fluorodeoxyglucose positron emission tomography-computed tomography (18F-FDG-PET/CT) in distinguishing PCNSL and HGG. According to their data, 18F-FDG-PET/CT had high accuracy for the differential diagnosis of HGG and PNCSL.

Artificial intelligence technologies, such as machine learning (ML), have improved radiomics predictive performance. Bahar et al. have systematically reviewed studies describing ML models for glioma grade prediction and evaluated the possibility of bringing ML from bench to clinic. The review highlighted that studies using ML applied to glioma had reported a high predictive accuracy. However, the authors have also demonstrated that to increase the standardization and the reproducibility of this technique in the glioma field, it is crucial to train and test on large, multi-institutional datasets, and adhere to reporting guidelines.

Anti-silencing function-1-B (ASF1B) belongs to the histone chaperone H3/H4 family. This protein is mainly involved in the regulation of cell proliferation and may be a potential prognostic marker in several tumors. Zhu H. et al. evaluated the role of *ASF1B* expression levels in gliomas. Transcriptomic clinical data were downloaded from three databases (genotypic tissue expression—GTEx, The Cancer Genome Atlas database—TCGA, and the Chinese Gliomas Genome Atlas database) and *ASF1B* levels were investigated in association with clinical variables. Authors found that high levels of *ASF1B* were associated with poor outcomes for glioma patients.

## Conclusions

Recently we have observed the advent of new advanced methodologies, bioinformatics, and genomic investigations; we are now witnessing the impact of these advances in the study of brain tumors. The collection of articles on this Research Topic encompasses the extent of attempts being done to advance scientific research, clinical diagnosis, and therapeutic development; these efforts are needed to obtain the aim of improving and lengthening the life of brain tumor patients.

## Author contributions

All authors listed have made a substantial, direct, and intellectual contribution to the work and approved it for publication.
